# Regulatory *VCAN* polymorphism is associated with shoulder pain and disability in breast cancer survivors

**DOI:** 10.1186/s40246-021-00337-0

**Published:** 2021-06-23

**Authors:** Trevor S. Mafu, Alison V. September, Delva Shamley

**Affiliations:** 1 Division of Exercise Science and Sports Medicine, Department of Human Biology, Faculty of Health Sciences, University of Cape Town, Cape Town, South Africa; 2 International Federation of Sports Medicine (FIMS) Collaborative Centre of Sports Medicine, ESSM, University of Cape Town, Cape Town, South Africa; 3 Clinical Research Centre, Department of Human Biology, Faculty of Health Sciences, University of Cape Town, Cape Town, South Africa; 4 Head of Division of Clinical Anatomy & Biological Anthropology, Department of Human Biology, Anatomy Building, Medical School, University of Cape Town, Cape Town, South Africa

**Keywords:** Proteoglycans, Variant, Connective tissue, Chronic pain, Shoulder dysfunction

## Abstract

**Background and purpose:**

Shoulder morbidity following breast cancer treatment is multifactorial. Despite several treatment- and patient-related factors being implicated, unexplained inter-individual variability exists in the development of such morbidity. Given the paucity of relavant genetic studies, we investigate the role of polymorphisms in candidate proteoglycan genes.

**Patients and methods:**

We conducted a cross-sectional study on 254 South African breast cancer survivors, to evaluate associations between shoulder pain/disability and ten single nucleotide polymorphisms (SNPs) within four proteoglycan genes: *ACAN* (rs1126823 G>A, rs1516797 G>T, rs2882676 A>C); *BGN* (rs1042103 G>A, rs743641 A>T, rs743642 G>T); *DCN* rs516115 C>T; and *VCAN* (rs11726 A>G, rs2287926 G>A, rs309559). Participants were grouped into no–low and moderate–high shoulder pain/disability based on total pain/disability scores: < 30 and ≥ 30, respectively using the Shoulder Pain and Disability Index (SPADI).

**Results:**

The GG genotype of *VCAN* rs11726 was independently associated with an increased risk of being in the moderate-to-high shoulder pain (P = 0.005, OR = 2.326, 95% CI = 1.259–4.348) or disability (P = 0.011, OR = 2.439, 95% CI = 1.235–4.762) categories, after adjusting for participants’ age. In addition, the T-T-G inferred allele combination of *BGN* (rs74364–rs743642)–*VCAN* rs11726 was associated with an increased risk of being in the moderate-to-high shoulder disability category (0 = 0.002, OR = 2.347, 95% CI = 1.215–4.534).

**Conclusion:**

Our study is first to report that *VCAN* rs11726, independently or interacting with *BGN* polymorphisms, is associated with shoulder pain or disability in breast cancer survivors. Whereas our findings suggest an involvement of proteoglycans in the etiology of shoulder pain/disability, further studies are recommended.

**Supplementary Information:**

The online version contains supplementary material available at 10.1186/s40246-021-00337-0.

## Introduction

Shoulder pain and disability are common chronic upper-limb morbidities among female breast cancer survivors [1, 2]. We recently reported that 74% and 62% of breast cancer survivors report some level of pain and functional disability, respectively, at least 1 year post-surgery [1]. The most recent systematic review of upper limb morbidity amongst breast cancer survivors by Hidding *et al*. [2] estimates prevalence of upper-limb pain and disability (reduced range of motion) at 9–68% and 6–31 %, respectively, beyond 1 year post-surgery. Shoulder pain and disability are strongly correlated [1], and may persist beyond 7 years following treatment [3]. Given that such morbidities have a negative impact on the quality of life of affected individuals [4], understanding the underlying etiology remains an urgent need.

The etiology underlying shoulder pain/disability remains poorly understood. Contributing treatment-related factors include type of breast surgery, type of axillary surgery and adjuvant therapy, with absolute risk increases of 1–21% for persistent pain [2, 5, 6]; patient-related factors include age, presence of preoperative pain, presence of acute post-operative pain and genetic predisposition, with absolute risk increases of 2–7% for persistent pain [6, 7]. Nonetheless, there remains a paucity of studies investigating the role of genetic factors in the interindividual variability in developing shoulder pain/disability amongst breast cancer survivors.

A growing body of evidence, from non-cancer-related conditions, supports the role of polymorphisms within genes encoding structural and regulatory extracellular matrix (ECM) proteins in modulating susceptibility to shoulder pathology [8–11]. Although no associations between polymorphisms in proteoglycan-encoding genes and non-cancer-related shoulder conditions have been reported to date, proteoglycans are important ECM components whose expression levels are altered in such conditions [12, 13]. In particular, changes in expression of the proteoglycans—aggrecan (ACAN), versican (VCAN), biglycan (BGN) and decorin (DCN)—have been reported in rotator cuff disease [12, 13]. Moreover, associations have been reported between proteoglycan gene polymorphisms and other connective tissue conditions such as anterior cruciate ligament ruptures [10, 14–16]. The hylectan proteoglycans, including ACAN and VCAN are important structural components in connective tissues such as tendons and ligaments. The small leucine-rich proteoglycans (SLRPs) including BGN and DCN regulate collagen fibrillogenesis, and are important modulators of the angiogenesis and the transforming growth factor beta (TGF-β) signaling pathways amongst others [17]. Given the role of proteoglycans in the ECM of connective tissues, functional polymorphisms in proteoglycan-encoding genes may, perhaps, lead to altered signaling and/or biomechanical properties in tissues such as tendons or ligaments of the shoulder. Indeed, studies on animal models have demonstrated that changes in expression of decorin and biglycan alter mechanical properties of tendons including failure load, stiffness, dynamic modulus and viscosity [18]. We hypothesize that polymorphisms in proteoglycan-encoding genes may be associated with shoulder pain/disability amongst breast cancer survivors. Our aim, therefore, was to investigate the association between candidate gene polymorphisms within proteoglycan-encoding genes and shoulder pain/disability following breast cancer treatment in women.

## Methods

### Study design

The study design is a pilot, cross-sectional, genetic association study based on the candidate gene approach.

### Participants and setting

A total of 254 participants were conveniently recruited in the year period 2013–2018, from the waiting room of the Oncology Clinic of a tertiary public teaching hospital in South Africa. All eligible participants (Table 1) agreeing to participate gave written informed consent. The recruited participants self-identified as ‘mixed-ancestry’ ethnicity, a rich genetic admixture ancestrally derived from immigrants from Western Europe, West Africa, Asia and the indigenous Southern African populations [19].

**Table 1:** Inclusion and exclusion criteria for participant recruitment

Inclusion criteria	Exclusion criteria
> 18 years old	History of shoulder or neck pathology prior to treatment for breast cancer
Females	Diagnosed connective tissue disorders such as rheumatoid arthritis or systemic lupus erythematosus
Unilateral breast cancer	Diagnosed renal insufficiency, diabetes mellitus or hyper-cholesterolemia
≥ 1 year after surgery	Diagnosed local recurrence
Self-declared ‘Mixed-ancestry’ ethnicity	Diagnosed lymphedema

### Study procedures

Study procedures have been previously reported [7]. Briefly, eligible consented participants completed the Shoulder Pain and Disability Index (SPADI) questionnaire and had their bloods drawn by venipuncture at the cubital fossa of the unaffected side using EDTA vacutainer tubes. Whole blood samples were immediately stored at − 20 °C until total DNA extraction using the method descried by Lahiri *et al.* [20]. Extracted DNA was stored long-term at − 20 °C. Relevant information for each participant including age, tumor grade, surgery data, and adjuvant therapy data were obtained from participants’ medical records.

### Patient-reported outcome measure

The primary outcome measure in this study was the SPADI, a validated and reliable patient-reported questionnaire with two domains: Pain (5 items) and Disability (8 items) [21, 22]. Participants rated pain or difficulty associated with specific activities of daily living on a visual analog scale (VAS) of 0 (no pain/difficulty) to 10 (extreme pain/difficulty). Symptom scores for both SPADI domains were reported as percentages of possible total scores [22].

Pain and disability scores were categorized according to score effects on activities of daily living and clinical relevance [7]; SPADI scores > 30 are regarded as having moderate–severe effects on activities of daily living [23], while patients with specific shoulder pain diagnoses, or on pain medication, were reported to have scores > 30 [21]. The reference ‘no–low’ category consisted of participants with SPADI pain/disability scores < 30, whereas the case ‘moderate–high’ category consisted of participants with SPADI pain/disability scores ≥ 30.

### Genetic variables

Exposures in this study were the total genotypes obtained from genotyping single nucleotide polymorphisms (SNPs) within four candidate proteoglycan genes: *ACAN* (rs1126823 A>G, rs1516797 T>G, rs2882676 A>C); *BGN* (rs1042103 G>A, rs743641 A>T, rs743642 G>T); *DCN* rs516115 C>T; and *VCAN* (rs11726 A>G, rs2287926 G>A, rs309559 A>G).

### Single-nucleotide polymorphism (SNP) selection

SNPs with global minor allele frequency > 0.15 in the ENSEMBL database (http://www.ensembl.org) were selected for investigation based on meeting one or more of the following criteria:

- Identified from a whole exome sequencing project on risk factors for tendinopathy or musculoskeletal soft tissue injuries [24]
- Functional significance, based on reported effects on gene expression or protein function
- Located in regulatory gene regions
- Previous associations with multifactorial soft-tissue shoulder conditions.

A total of ten SNPs within four proteoglycan-encoding genes were included (Tables 4 and 5). In order to ensure robust genetic association analyses, only SNP call rates of > 95% and Hardy–Weinberg *p* values > 0.05 were included.

### Genetic analyses

Genotyping was performed using TaqMan™ assays (Applied Biosystems) in 96-well plates, following manufacturer’s instructions in a QuantStudio™ 3 Real-Time PCR System (Applied Biosystems) at the Division of Exercise Science and Sports Medicine, University of Cape Town. Both negative controls (no DNA sample), positive controls (DNA of known genotypes) and replicates (sample duplicates) were included in every plate to evaluate the reliability of the PCR and detect potential genotyping errors. The genotyping data were analyzed on Thermo Fisher Cloud genotyping analysis Software Version: 3.3.0-SR2-build 21 with automatic genotype calling for the 9 SNPs: *ACAN* (rs1126823 A>G, rs1516797 T>G); *BGN* (rs1042103 G>A, rs743641 A>T, rs743642 G>T), *DCN* (rs516115 C>T) and *VCAN* (rs11726 A>G, rs2287926 G>A, rs309559 A>G). Due to less-efficient amplification for the *ACAN* rs2882676 A>C SNP, genotypes were manually called and compared with the manual calls of an independent blinded technical support member with 99.7% similarity.

### Bias

Nine percent (23 out of 254) of participants could not provide bloods because they were lost after consent when they went for further medical examination in the clinic. Although there may be differences between participants who provided blood and those who did not, it is unlikely as all participants were randomly identified and consented.

### Statistical analysis

The calculation of sample size for this study, using QUANTO version 1.2.469 [25], was described previously [7]. A sample size of *N* = 231 was regarded likely sufficient to detect odds ratios of ≥ 2.5 for allele frequencies ≥ 0.15, assuming an expected average baseline risk for shoulder pain (32%) and disability (25%), for dominant or additive genetic models [7].

Demographic and clinical data were analyzed using Statistica version 13.2.70 [26]. Mann Whitney U tests were used to evaluate differences in quantitative characteristics between the shoulder pain/disability categories, given that the data was non-parametric. Fisher’s exact and Chi-square analyses were performed to evaluate differences in categorical demographic and clinical characteristics between the shoulder pain/disability categories.

The genotype data were analyzed using R Studio version 1.3.895 running R version 3.6.3 [27, 28]. Chi-square and Fisher’s exact tests were used to evaluate differences in the genotype, allele and inferred haplotype frequencies between the shoulder pain/disability categories. Hardy–Weinberg equilibrium (HWE) and linkage disequilibrium (LD) were calculated using R package ‘genetics’ version 1.3.8.1.2 [29]. Logistic regression analyses were performed using R package ‘SNPassoc’ version 1.9-2 to evaluate the association between SNP genotype and shoulder pain/disability category membership [30]; the best model (with the lowest Akaike Information Criterion (AIC)) was chosen among dominant, recessive and log-additive models. Using the R package ‘haplo.stats’ Version 1.7.9 [31], inferred haplotypes for the *ACAN*, *BGN* and *VCAN* polymorphisms were constructed using the genotype date for each SNP investigated. To investigate possible gene–gene interactions in modulating risk for shoulder pain/disability, inferred allele combinations were constructed using the relevant genotype data for the genes. The choice of SNPs for inferred allele combination construction was based on stepwise backward elimination logistic regression analysis. In each step, the least informative SNPs whose exclusion lowered, and therefore improved, the AIC of the model was removed until the last three SNPs representing the best model for shoulder pain or disability with three SNPs. To avoid saturating the models while controlling for confounding, only participants’ age, which was shown to be associated with our primary outcomes, was included in all multivariate regression models. For all inferred haplotypes or allele combinations, a low haplotype frequency cut-off of 4% was used to improve validity. Stepwise regression analyses were performed using R package ‘MASS’ version 7.3-51.5 [32]. R package ‘ggplot2’ version 3.3.2 was used to produce all graphs [33]. The level of significance was set as *p* < 0.05.

## Results

### Differences in clinical and demographic characteristics between pain/disability categories

Participants in the moderate–high shoulder pain category were significantly younger compared with those in the no–low shoulder pain category 53.8 (45.3–64.3) vs. 60.8 (53.5–65.5), *p* = 0.001) (Table 2). Similarly, participants in the moderate–high shoulder disability category were significantly younger compared with those in the no–low disability category (54.4 (45.0–64.9) vs. 60.4 (53.2–65.2), *p* = 0.014) (Table 3). However, no significant differences (*p* > 0.05) were noted between participants in the no–low and moderate–high shoulder pain/disability categories for all other variables. Despite being statistically insignificant, a lower proportion of participants in the moderate–high shoulder pain/disability category underwent the more aggressive surgeries: mastectomy and axillary lymph not dissection, compared with those in the no–low shoulder pain/disability category (Tables 2 and 3). In addition, a higher proportion of participants in the moderate–high shoulder pain/disability category received adjuvant chemotherapy compared with those in the no–low shoulder pain/disability category (Tables 2 and 3). Receipt of adjuvant radiotherapy was only notable for shoulder pain categories, with a higher proportion of participants in the moderate–high category receiving the same compared with participants in the no–low category (Table 2).

**Table 2:** Differences in demographic and clinical characteristics between shoulder pain categories

***Characteristic***	***Level/unit***	***No–low*** ***(n = 183)***	***Moderate–high (n = 71)***	***p value***	***Test***
***Age at consent***	Years	60.8 (53.5–65.5)	53.8 (45.3–64.3)	**0.001**	MU
***Time after surgery***	Years	3.0 (1.8–4.7)	2.3 (1.7–4.0)	0.139	MU
***Nodes removed***		10.0 (5.5–15.0)	9.0 (5.0–12.0)	0.154	MU
***Side of primary***	Left Right	52 (94) 48 (87)	54 (38) 46 (32)	0.779	F
***Tumor grade***	III II I	24 (38) 51 (81) 26 (41)	23 (15) 53 (34) 23 (15)	0.930	χ^2^
***Type of surgery***	Mastectomy WLE	81 (148) 19 (34)	72 (50) 28 (19)	0.165	F
***Lymph node surgery***	ALND SLNB	85 (153) 15 (28)	78 (54) 22 (15)	0.263	F
***Chemotherapy***	Yes No	74 (131) 26 (46)	84 (58) 16 (11)	0.129	F
***Hormonal therapy***	Yes No	77 (134) 23 (39)	79 (54) 21 (14)	0.863	F
***Hormonal regimen***	Tamoxifen	62 (106)	60 (41)	0.238	χ^2^
	Aromatase inhibitor	8 (14)	4 (3)		
	Both	7 (12)	15 (10)		
***Radiotherapy***	Yes No	68 (114) 32 (53)	74 (50) 26 (18)	0.531	F

Notes: Data presented as medians with interquartile ranges in parentheses, or % frequencies with actual counts (n) in parentheses. *p* values in bold typeset indicate statistical significance (*p* < 0.05).

Abbreviations: *WLE,* wide local excision; *ALND,* axillary lymph node dissection; *SLNB*, sentinel lymph node biopsy; *MU,* Mann Whitney U test; *F,* Fisher’s exact test; χ2, Chi-squared test

**Table 3:** Differences in demographic and clinical characteristics between shoulder disability categories

***Characteristic***	***Level/unit***	***No–low*** ***(n = 206)***	***Moderate–high*** ***(n = 48)***	***p value***	***Test***
***Age at consent***	Years	60.4 (53.2–65.2)	54.4 (45.0–64.9)	**0.014**	MU
***Time after surgery***	Years	2.9 (1.7–4.5)	2.6 (1.8–4.3)	0.728	MU
***Nodes removed***		10.0 (5.0–14.0)	9.0 (6.0–13.0)	0.630	MU
***Side of primary***	Left Right	52 (106) 48 (97)	54 (26) 46 (22)	0.873	F
***Tumor grade***	III II I	23 (41) 53 (96) 24 (44)	28 (12) 44 (19) 28 (12)	0.572	χ^2^
***Type of surgery***	Mastectomy WLE	79 (162) 21 (42)	77 (36) 23 (11)	0.693	F
***Lymph node surgery***	ALND SLNB	84 (170) 16 (33)	79 (37) 21 (10)	0.398	F
***Chemotherapy***	Yes No	76 (150) 24 (48)	81 (39) 19 (9)	0.567	F
***Hormonal therapy***	Yes No	78 (151) 22 (43)	79 (37) 21 (10)	1.000	F
***Hormonal regimen***	Tamoxifen	62 (119)	60 (28)	0.452	χ^2^
	Aromatase inhibitor	8 (15)	4 (2)		
	Both	8 (15)	15 (7)		
***Radiotherapy***	Yes No	71 (134) 29 (56)	67 (30) 33 (15)	0.594	F

Notes: Data presented as medians with interquartile ranges in parentheses, or % frequencies with actual counts (n) in parentheses. p values in bold typeset indicate statistical significance (*p* < 0.05).

Abbreviations: *WLE,* wide local excision; *ALND*, axillary lymph node dissection; *SLNB*, sentinel lymph node biopsy; *MU*, Mann Whitney U test; *F*, Fisher’s exact test; *χ*^*2*^, Chi-squared test.

Interestingly, we noted that (*p* = 0.014) a higher proportion of participants with the GG (88.2%, *n* = 60) genotype for *ACAN* rs1126823 A>G received hormonal therapy compared with those with AA or AG (73.0%, *n* = 111) genotypes (Supplementary Table 1). A significantly (*p* = 0.034) lower proportion of participants with the TT (58.3%, *n* = 14) genotype for *BGN* rs743641 A>T received hormonal therapy compared with participants with AA or AT (80.1%, *n* = 157) genotypes. Furthermore, a significantly (*p* = 0.001) higher proportion of participants with the AA (92.9%, *n* = 65) genotype of *ACAN* rs2882676 A>C had mastectomy compared with those with AC or CC (73.1%, *n* = 114) genotypes. Whereas, individuals with a TT (66.7%, *n* = 44) genotype for *DCN* rs516115 C>T were significantly (*p* = 0.007) less likely to have mastectomy compared with CT or CC (84.0%, *n* = 136) genotype carries.

### Genotype/allele frequency distributions between shoulder pain/disability categories

The genotype frequencies of the *VCAN* rs11726 A>G polymorphism were significantly different (*p* < 0.05) between the no–low and moderate–high categories for both shoulder pain and disability, including after adjustment for participants’ age (Tables 4 and 5). In particular, the GG genotype of *VCAN* rs11726 A>G was significantly more common (*p* = 0.005; OR = 2.326, 95% CI = 1.250–4.348) in the moderate–high shoulder pain category (48%) in comparison with the no–low shoulder pain category (29%) (Table 4). Similarly, the GG genotype of *VCAN* rs11726 A>G was significantly more common (*p* = 0.011; OR = 2.439, 95% CI = 1.235–4.762) in the moderate–high shoulder disability category (51%) in comparison with the no–low shoulder disability category (30%) (Table 5). No significant differences were noted in allele frequency distributions between the no–low and moderate–high categories for the *VCAN* rs11726 A>G polymorphism (Tables 4 and 5). However, there was a trend (p = 0.069) towards over-representation of the A allele of *VCAN* rs11726 A>G in the no–low shoulder disability category (44%) in comparison with the moderate–high disability category (33%) (Table 5).

**Table 4:** Genotype/minor-allele frequency distributions of the *ACAN*, *BGN*, *DCN* and *VCAN* polymorphisms for shoulder pain categories

Gene	SNP	Genotype or allele	No–low (*n* = 170)	Moderate–high (*n* = 61)	*p* value
***ACAN***	rs1126823 A>G	G/G	29 (50)	33 (20)	0.609 (0.459)
		A/G	45 (76)	47 (29)	
		A/A	26 (44)	20 (12)	
		A	48 (164)	43 (53)	0.398
	rs1516797 T>G	G/G	28 (47)	29 (18)	0.856 (0.959)
		G/T	44 (75)	46 (28)	
		T/T	28 (48)	25 (15)	
		T	50 (171)	48 (58)	0.673
	rs2882676 A>C	A/A	32 (54)	27 (16)	0.724 (0.591)
		A/C	46 (78)	48 (29)	
		C/C	22 (37)	25 (15)	
		C	45 (152)	49 (59)	0.456
***BGN***	rs1042103 G>A	G/G	74 (125)	70 (43)	0.350 (0.766)
		A/G	24 (41)	23 (14)	
		A/A	2 (4)	7 (4)	
		A	14 (49)	18 (22)	0.380
	rs743641 A>T	A/A	48 (82)	46 (28)	0.801 (0.763)
		A/T	42 (71)	41 (25)	
		T/T	10 (17)	13 (8)	
		T	31 (105)	34 (41)	0.573
	rs743642 G>T	G/G	54 (92)	52 (32)	0.476 (0.451)
		G/T	39 (67)	36 (22)	
		T/T	7 (11)	12 (7)	
		T	26 (89)	30 (36)	0.478
***DCN***	rs516115 C>T	T/T	29 (49)	30 (18)	0.684 (0.572)
		C/T	53 (90)	48 (29)	
		C/C	18 (31)	23 (14)	
		C	45 (152)	47 (57)	0.751
***VCAN***	rs11726 A>G	G/G	29 (49)	48 (29)	**0.004 (0.005)**
		A/G	55 (94)	31 (19)	
		A/A	16 (27)	21 (13)	
		A	44 (148)	37 (45)	0.239
	rs2287926 G>A	G/G	66 (112)	59 (36)	0.398 (0.374)
		A/G	29 (49)	38 (23)	
		A/A	5 (9)	3 (2)	
		A	20 (67)	22 (27)	0.601
	rs309559 A>G	G/G	26 (44)	29 (18)	0.863 (0.877)
		A/G	49 (83)	46 (28)	
		A/A	25 (42)	25 (15)	
		A	49 (167)	48 (58)	0.752

Notes: Genotype and allele frequencies are expressed as a percentage with the number of participants (n) in parentheses. p values in bold typeset indicate significance (*p* < 0.05), whereas p values in parentheses are adjusted for participants’ age at consent.

Abbreviations: *ACAN*, *Aggrecan*; *BGN*, *Biglycan*; *DCN*, *Decorin*; *VCAN*, *Versican*.

**Table 5:** Genotype/minor-allele frequency distributions of the *ACAN*, *BGN*, *DCN* and *VCAN* polymorphisms for shoulder disability categories

Gene	SNP	Genotype or allele	No–low (*n* = 188)	Moderate–high (*n* = 43)	*p* value
***ACAN***	rs1126823 A>G	G/G	30 (56)	33 (14)	0.870 (0.987)
		A/G	46 (87)	42 (18)	
		A/A	24 (45)	26 (11)	
		A	47 (177)	47 (40)	1.000
	rs1516797 T>G	G/G	29 (54)	26 (11)	0.864 (0.802)
		G/T	45 (84)	44 (19)	
		T/T	27 (50)	30 (13)	
		T	49 (184)	52 (45)	0.633
	rs2882676 A>C	A/A	31 (58)	29 (12)	0.895 (0.861)
		A/C	46 (86)	50 (21)	
		C/C	23 (43)	21 (9)	
		C	46 (172)	46 (39)	1.000
***BGN***	rs1042103 G>A	G/G	73 (137)	72 (31)	0.416 (0.401)
		A/G	25 (46)	21 (9)	
		A/A	3 (5)	7 (3)	
		A	15 (56)	17 (15)	0.619
	rs743641 A>T	A/A	49 (92)	42 (18)	0.431 (0.442)
		A/T	42 (78)	42 (18)	
		T/T	10 (18)	16 (7)	
		T	30 (114)	37 (32)	0.247
	rs743642 G>T	G/G	54 (102)	51 (22)	0.101 (0.101)
		G/T	40 (75)	33 (14)	
		T/T	6 (11)	16 (7)	
		T	26 (97)	33 (28)	0.226
***DCN***	rs516115 C>T	T/T	29 (54)	30 (13)	0.976 (0.888)
		C/T	52 (97)	51 (22)	
		C/C	20 (37)	19 (8)	
		C	46 (171)	44 (38)	0.905
***VCAN***	rs11726 A>G	G/G	30 (56)	51 (22)	**0.024 (0.033)**
		A/G	53 (99)	33 (14)	
		A/A	18 (33)	16 (7)	
		A	44 (165)	33 (28)	0.069
	rs2287926 G>A	G/G	67 (125)	54 (23)	0.113 (0.118)
		A/G	28 (53)	44 (19)	
		A/A	5 (10)	2 (1)	
		A	19 (73)	24 (21)	0.301
	rs309559 A>G	G/G	27 (51)	26 (11)	0.662 (0.685)
		A/G	49 (92)	44 (19)	
		A/A	24 (44)	30 (13)	
		A	48 (180)	52 (45)	0.550

Notes: Genotype and allele frequencies are expressed as a percentage with the number of participants (n) in parentheses. *p* values in bold typeset indicate significance (*p* < 0.05), whereas p values in parentheses are adjusted for participants’ age at consent.

Abbreviations: *ACAN*, *Aggrecan*; *BGN*, *Biglycan*; *DCN*, *Decorin*; *VCAN*, *Versican*.

For both shoulder pain and shoulder disability, no significant differences (*p* > 0.05) in the genotype/allele frequency distributions were noted between the no–low and moderate–high categories for the following SNPs: *ACAN* rs1126823 A>G, *ACAN* rs1516797 T>G, *ACAN* rs2882676 A>C, *BGN* rs1042103 G>A, *BGN* rs743641 A>T, *BGN* rs743642 G>T, *DCN* rs516115 C>T, *VCAN* rs2287926 G>A and *VCAN* rs309559 A>G (Tables 4 and 5). The genotype distributions for the whole group were in Hardy–Weinberg equilibrium (HWE exact test *p* > 0.05) for all SNPs investigated in this study (Supplementary Table 1).

### Inferred haplotype frequency distributions between shoulder pain/disability categories

**Fig. 1:**
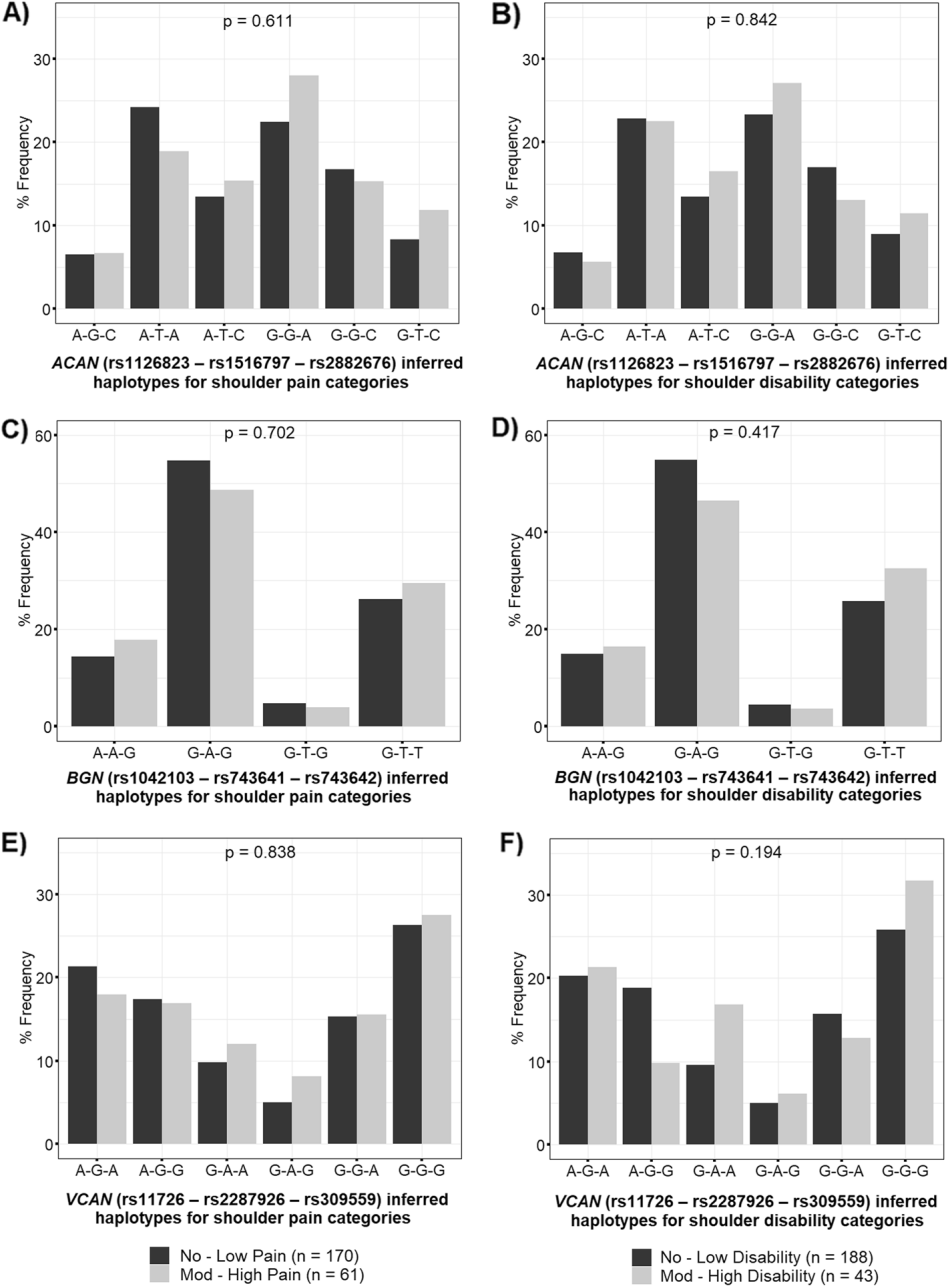
Frequency distributions of the *ACAN*, *BGN* and *VCAN* inferred haplotypes between shoulder pain/disability categories. Distributions of the (A and B) *ACAN* (rs1126823 G>A – rs1516797 G>T – rs2882676 A>C), (C and D) *BGN* (rs1042103 G>A – rs743641 A>T – rs743642 G>T) and (E and F) *VCAN* (rs11726 A>G – rs2287926 G>A – rs309559 A>G) inferred haplotypes between participants with no–low and moderate–high shoulder pain/disability following breast cancer treatment. Global p values (adjusted for participants’ age) are noted centrally at the top of each graph

No significant differences were noted in the frequency distribution of the *ACAN* (rs1126823 A>G – rs1516797 T>G – rs2882676 A>C), *BGN* (rs1042103 G>A – rs743641 A>T – rs743642 G>T) or *VCAN* (rs11726 A>G – rs2287926 G>A – rs309559 A>G) inferred haplotypes between the no–low and moderate–high shoulder pain/disability categories (p > 0.05) (Fig. 1).

### Inferred allele combination frequency distributions between shoulder pain/disability categories

**Fig. 2:**
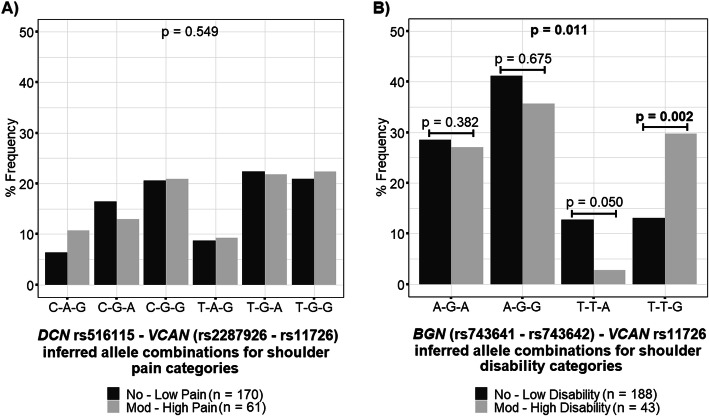
Frequency distributions of selected inferred allele combinations between shoulder pain/disability categories. Distributions of the A *DCN* rs516115 C>T – *VCAN* (rs2287926 A>C – rs11726 A>G) and B *BGN* (rs743641 A>T – rs743642 G>T) – *VCAN* rs11726 A>G inferred allele combinations between participants with no–low and moderate–high shoulder pain/disability following breast cancer treatment. Global p values are noted centrally at the top of each graph, while post hoc p values are noted just above each pair of bars. The p values in bold typeset indicate statistical significance

No significant differences (*p* > 0.05) were noted in the frequency distribution of the inferred *DCN* rs516115 C>T – *VCAN* (rs2287926 A>C – rs11726 A>G) allele combinations between no–low and moderate–high shoulder pain categories (Fig. 2A). However, significant differences were noted in the frequencies of the *BGN* (rs743641 A>T – rs743642 G>T) – *VCAN* rs11726 A>G (*p* = 0.011) inferred allele combinations between participants with no–low and moderate–high shoulder disability (Fig. 2B). In particular, the T-T-G inferred allele combination of *BGN* (rs743641 A>T–rs743642 G>T) – *VCAN* rs11726 A>G was significantly over-represented (*p* = 0.002; OR = 2.347, 95% CI = 1.215–4.534) in the moderate–high shoulder disability category compared with the no–low shoulder disability category (Fig. 2B). Moreover, a trend was noted towards over-representation (*p* = 0.050) of the T-T-A allele combination of *BGN* (rs743641 A>T – rs743642 G>T) – *VCAN* rs11726 A>G in the no–low shoulder disability category in comparison with the moderate-to-high disability category (Fig. 2B).

## Discussion

There is a paucity of studies investigating the role of genetic factors in modulating susceptibility to shoulder pain or disability amongst breast cancer survivors. Whereas proteoglycan gene polymorphisms or expression levels have been implicated in connective tissue conditions of the shoulder or other sites such as the knee [10, 12–16], their role in shoulder morbidity amongst breast cancer survivors is unknown. We found associations between *VCAN* rs11726 A>G genotype and *BGN* (rs743641 (251A>T) – rs743642 (318G>T)) – *VCAN* rs11726 (1429A>G) inferred allele combinations, and shoulder pain/disability among women following breast cancer treatment. To the best of our knowledge, our study is the first to report associations between proteoglycan gene polymorphisms and shoulder pain/disability amongst breast cancer survivors. This adds to the body of evidence indicating genetic predisposition to shoulder pain or disability following breast cancer treatment [7].

The *VCAN* rs11726 (1429A>G) polymorphism—associated with shoulder pain/disability both independently and in an inferred allele combination with *BGN*: rs743641 (251A>T) and rs743642 (318G>T)—is located in the 3' UTR gene region and has been shown to alter *VCAN* gene expression levels (Ensembl genome browser data, database version 100.38, Genome Reference Consortium Human Build 38) [34]. In particular, the G allele of *VCAN* rs11726 demonstrates higher levels of expression relative to the ancestral A allele in skeletal muscle and pancreatic cells [34]. The *VCAN* rs11726 polymorphism is also located within the long non-coding transcript, *VCAN-AS1*, which is an antisense RNA transcript for *VCAN* and hence another possible mechanism for regulating *VCAN* expression. Although non-significant, it was interesting to observe a trend (*p* = 0.069) towards under-representation of the G allele amongst participants reporting no–low levels of shoulder disability compared with those that reported moderate–high levels of shoulder disability (Table 5). Increasing the samples population is therefore important towards evaluating the association of these alternate alleles with shoulder disability. The exact mechanism by which the *VCAN* rs11726 polymorphism may lead to shoulder disability or pain is not clear, given the versatile and complex functions of VCAN [17]. Possibly, VCAN interacts with important signaling factors such as the fibrogenesis factor TGF-β and the inflammatory factor nuclear factor kappa B (NF-κB), which may contribute to shoulder morbidity by promoting fibrogenesis and nociceptive signaling, respectively [17, 35, 36]; perhaps, increased expression of *VCAN* amongst G allele carriers leads to enhanced fibrosis and pain signaling in the shoulder in response to late treatment effects amongst breast cancer survivors. The *BGN* rs743641 and rs743642 polymorphisms are both located in the 3' UTR gene regulatory region, but have no reported gene expression correlations nor previous associations with connective tissue disorders of the shoulder. Another possible explanation for the role of the associated *VCAN* and *BGN* polymorphisms in this study could be that they are in linkage disequilibrium with other SNPs that are involved in the development of pain or disability. Whereas no previous associations have been reported between *VCAN* rs11726, *BGN* rs743641 or rs743642, and shoulder morbidity to date, other polymorphisms in *BGN* have been implicated in connective tissue disorders such as ACL ruptures [14, 16]. In addition, upregulation of *VCAN* and *BGN* expression has been demonstrated in a rat model of rotator cuff injury [37].

Consistent with previous reports on upper limb morbidity amongst breast cancer survivors [3, 6, 38], participants’ age was significantly associated with both shoulder pain and shoulder disability following breast cancer treatment in our study. Younger participants were more likely to be in the moderate–high shoulder pain or disability category. The link between age and pain reporting remains unclear. One possible explanation is the reported reduction in pain sensitivity with age as determined from pressure pain threshold (PPT) measurements which may be relevant for movement-related pain that is measured by the SPADI instrument in our study [39]. Given that PPT measurements are subjective, the reported association may reflect changes in pain perception with age. Contrary to previous reports [2, 5, 6, 40, 41], type of breast surgery, having axillary surgery and receipt of adjuvant therapy were not significantly associated with shoulder pain or shoulder disability in our study. In fact, a higher frequency of the more aggressive surgical procedures mastectomy and ALND—compared with the conservative WLE and SLNB—was observed in the no–low pain/disability group (Tables 2 and 3). This finding may perhaps be specific to our cohort; pain reporting has been associated with ethnicity [1], and the average time after treatment in our cohort is longer than that of most similar studies. Consistent with our findings, De Groef *et al*. [42] reported a high prevalence of upper limb morbidity amongst breast cancer patients who underwent the less invasive sentinel node-negative suggesting that upper limb morbidity amongst breast cancer survivors may not be largely explained by factors related to surgical management after long follow-up periods.

It was interesting to note the genotype associations of *ACAN* rs1126823 G>A, *ACAN* rs2882676 A>C, *BGN* rs743641 A>T and *DCN* rs516115 C>T with treatment characteristics in our cohort. This may reflect, at least in part, the role of proteoglycans in the development and progression of cancer, thereby, influencing treatment options and selections. Although, no studies to date have demonstrated their roles in breast cancer, *BGN* and *DCN* have been implicated in the development and progression of endometrial, bladder, colon, blood or lung cancers [43].

### Limitations

Despite being adequate for medium–large effect sizes (OR ≥ 2.0), our sample size was largely underpowered (power < 80%) for small effect sizes (OR = 1.5). As a result, we did not adjust for multiple comparisons based on the number of SNPs (familywise error rate), given its exploratory nature. Larger sample sizes may detect significant differences in other clinical/treatment characteristics and genotype/allele distributions included in this study. Although clinical relevance was used in creating shoulder pain/disability categories, there was no wide score gap between them. Therefore, close to the boundary score of 30, individuals with otherwise similar shoulder pain/disability characteristics may be in different categories. Ethnicity was determined by self-report, a less reliable method than genetic ancestry estimates, and therefore, there is a possibility of undetermined population stratification in our sample. While determination of genetic ancestry is very useful in detecting population stratification, this study only tested targeted loci using functional polymorphisms in a hypothesis-driven approach. Applying genetic ancestry estimates in this case would be a completely different study, much larger than the one described in this manuscript, such as a genome-wide association study. Lastly, associations between SNPs in *ACAN*/*BGN*/*DCN* genes and treatment characteristics (Supplementary Table 1) may have an undetermined influence on our findings and should be explored in greater depth with breast cancer risk.

## Conclusion

Our findings provide evidence of association between polymorphisms in proteoglycan-encoding genes and shoulder pain/disability among women following breast cancer treatment. Future studies in independent populations with larger sample sizes are warranted to replicate our findings and further characterize the reported associations.

## Supplementary Information

**Additional file 1: Supplementary table 1.** *P*-values for tests on Hardy–Weinberg equilibrium and Genotype effects on descriptive characteristics

## Data Availability

The datasets used and/or analysed during the current study are available from the corresponding author on reasonable request.
