# Supplementary material for: Regulatory VCAN polymorphism is associated with shoulder pain and disability in breast cancer survivors
Source: Hum Genomics. 2021 Jun 23;15:36. doi: 10.1186/s40246-021-00337-0 (PMC8220713; doi:10.1186/s40246-021-00337-0)
Supplement: Supplementary file 1 — Additional file 1: Supplementary table 1. P-values for tests on Hardy–Weinberg equilibrium and Genotype effects on descriptive characteristics [file 40246_2021_337_MOESM1_ESM.docx]

**Supplementary table 1:** P-values for tests on Hardy-Weinberg equilibrium and Genotype effects on descriptive characteristics

| **SNP** | **HWE** | **Time after surgery** | **Nodes removed** | **Side of primary** | **Tumor grade** | **Type of surgery** | **Axillary surgery** | **Chemotherapy** | **Hormonal therapy** | **Radiotherapy** |
| --- | --- | --- | --- | --- | --- | --- | --- | --- | --- | --- |
| ***ACAN*** |  |  |  |  |  |  |  |  |  |  |
| rs1126823 A>G | 0.189 | 0.704 | 0.322 | 0.441 | 0.613 | 0.145 | 0.352 | 0.462 | **0.043** | 0.863 |
| rs1516797 T>G | 0.090 | 0.779 | 0.319 | 0.902 | 0.050 | 0.544 | 0.671 | 0.921 | 0.863 | 0.462 |
| rs2882676 A>C | 0.509 | 0.789 | 0.387 | 0.960 | 0.698 | **0.002** | 0.069 | 0.205 | 0.369 | 0.383 |
| ***BGN*** |  |  |  |  |  |  |  |  |  |  |
| rs1042103 G>A | 0.322 | 0.241 | 0.068 | 0.707 | 0.313 | 0.102 | 0.293 | 0.433 | 0.067 | 0.744 |
| rs743641 A>T | 0.547 | 0.903 | 0.241 | 0.713 | 0.202 | 0.720 | 0.326 | 0.870 | **0.045** | 0.828 |
| rs743642 G>T | 0.740 | 0.990 | 0.789 | 0.599 | 0.224 | 0.729 | 0.678 | 0.865 | 0.138 | 0.712 |
| ***DCN*** |  |  |  |  |  |  |  |  |  |  |
| rs516115 C>T | 0.597 | 0.624 | 0.332 | 0.454 | 0.389 | **0.009** | 0.090 | 0.537 | 0.845 | 0.468 |
| ***VCAN*** |  |  |  |  |  |  |  |  |  |  |
| rs11726 A>G | 0.893 | 0.484 | 0.408 | 0.770 | 0.392 | 0.787 | 0.691 | 0.945 | 0.695 | 0.079 |
| rs2287926 G>A | 0.548 | 0.141 | 0.681 | 0.131 | 0.172 | 0.289 | 0.823 | 0.918 | 0.523 | 0.967 |
| rs309559 A>G | 0.512 | 0.770 | 0.134 | 0.209 | 0.392 | 0.668 | 0.160 | 0.622 | 0.770 | 0.322 |
| **Notes**: P-values for HWE and tumor grade were obtained from Chi square tests; P-values for side of primary cancer and type of treatment received were obtained from Fisher’s exact tests; P-values for participants’ age at consent, time after surgery and number of lymph nodes removed were obtained from Kruskal-Wallis tests. Bold typeset p-values indicate significance **(p<0.05)**.  **Abbreviations**: SNP, single nucleotide polymorphism; *ACAN*, *Aggrecan*; *BGN*, *Biglycan*; *DCN*, *Decorin*; *VCAN,* *Versican*; HWE, Hardy-Weinberg equilibrium. | | | | | | | | | | |
